# Transitions across states with and without difficulties in performing activities of daily living and death: a longitudinal comparison of ten European countries

**DOI:** 10.1007/s10433-023-00763-0

**Published:** 2023-05-18

**Authors:** Alejandra Marroig

**Affiliations:** Instituto de Estadística, Universidad de la República, Montevideo, Uruguay

**Keywords:** Ageing, Care dependency, Activities of daily living, Multi-state models, Longitudinal analysis, SHARE

## Abstract

Ageing has been related to the onset of disability and dependency in older adults. There is a need to better understand the disability and dependency trajectories of older adults and their relationship with socio-demographic characteristics and institutional or cultural context. This study analyses the role of age, sex, education and self-perceived health in disability, dependency and death transitions, addressing the heterogeneity across European countries and inconsistencies when using different measures of disability. Multi-state models were adjusted to evaluate the role of risk and protective factors in the transitions to disability, dependency and death. Difficulties in performing activities of daily living (ADLs) assess disability and dependency states. Data were from the Survey of Health, Ageing and Retirement in Europe conducted in 2004–2013, considering individuals aged 65 and older at baseline from Austria, Belgium, Denmark, France, Germany, Italy, the Netherlands, Spain, Sweden and Switzerland. The results showed that transitions to disability and dependency varied with age, sex, education and self-perceived health. The probability of transition to disability and dependency states increases until the age of 70 for all countries. However, there was heterogeneity in the disability and dependency trajectories with ageing between men and women. In most countries, women live with difficulties and may need help for longer than men. Care policies should consider sex differences to decrease the burden of care of informal caregivers, particularly in countries where care systems are absent or partially developed and a high level of family obligations to care needs exist.

**Supplementary Information:**

The online version contains supplementary material available at 10.1007/s10433-023-00763-0.

## Introduction

Ageing has been related to the onset of disability and care dependence in older adults, and help from others may be needed daily (Barnay and Juin 2016; Navarro Espigares et al. 2008; Van Houtven and Norton 2004). Some individuals start having difficulties and may require help earlier in life, while others may preserve independence for longer. The different disability and care dependency trajectories with ageing could vary according to socio-demographic characteristics and may be related to the institutional or cultural context, the type of help needed, the met or unmet care needs, and their quality of life (Abdi et al. 2019; Geerts and Van den Bosch 2012).

Disability and care dependence are complex and multidimensional constructs related to health conditions and contextual factors. Disability is an umbrella term for impairments, activity limitations and participation restrictions, and represents the negative aspects of the interaction between the individual's health condition and personal and environmental factors (WHO 2001). Meanwhile, care dependence is defined as the need for human help or care beyond that habitually required (Harwood et al. 2004). In previous works, the activities of daily living (ADLs) were used to operationalise these constructs (Amengual et al. 2021; Carmona-Torres et al. 2019; Edjolo et al. 2016; Jerez-Roig et al. 2018; Lima et al. 2018; Millán-Calenti et al. 2010; Rodríguez-Sampayo et al. 2011; Scheel-Hincke et al. 2020; WHO 2015). Typically, ADLs are classified into Basic Activities of Daily Living (BADLs) and Instrumental Activities of Daily Living (IADLs), thus representing the diversity and multidimensionality of the constructs (Katz et al. 1963; Lawton and Brody 1969). The ability to perform BADLs assesses functional capacity related to the individual's physical condition, whereas the IADLs evaluate activities that are more cognitively demanding and often approximate the potential of older adults to live independently (Millán-Calenti et al. 2010). Recently, Advanced Activities of Daily Living (AADLs), including social and community participation, the ability to communicate, and making decisions, have been also considered (WHO 2001).

The health status of older adults is characterised by the interaction of comorbidity, disability and frailty, which are also related to the use of health and social services, as older adults may need help with activities of daily living (Fried et al. 2004). Increasing age is associated with a higher risk of disability and need for help (care dependency), while sex, education and health status emerged as stratifying factors for disability and need for help (Arrighi et al. 2017; Barnay and Juin 2016; Bora and Saikia 2015; Cameron et al. 2010; Carmona-Torres et al. 2019; Crimmins et al. 2011). A higher educational attainment is associated with better health status as it is associated with better social and economic conditions (Hoogendijk et al. 2014). Health conditions such as dementia, stroke, limb impairment and depression are key contributors to disability and care dependence (Prina et al. 2020) and self-perceived health has been reported as the most informative measure of health status predicting mortality in population studies (Jylhä 2009). Moreover, hypertension confers a greater risk for ischemic stroke among women compared with men and after nonfatal stroke women have greater disability than men (Mokdad et al. 2018; Wenger et al. 2016). Therefore, it is relevant to evaluate how sex, education and self-perceived health shape the disability trajectories of older adults.

Previous studies stated that women outlive men with poorer health conditions, which is a well-documented phenomenon known as the “male–female health-survival paradox” (Austad 2006; Lima et al. 2018; Oksuzyan et al. 2008; Thorslund et al. 2013). Some studies looked at sex differences in performing ADLs (Crimmins et al. 2011; Lima et al. 2018; Millán-Calenti et al. 2010; Scheel-Hincke et al. 2020). In particular, Scheel-Hincke et al. (2020) analysed sex differences in performing ADLs using a large pooled cross-sectional setting for middle-aged and older adults who participated in the Survey of Health, Ageing, and Retirement in Europe (SHARE). They reported heterogeneity in the differences between men and women across European regions. Moreover, they argued that a widening of the sex gap with increasing age may be consistent with a survival effect, which leaves the healthiest men in the sample (Scheel-Hincke et al. 2020).

This study aims to evaluate whether transitions towards states with difficulties in performing ADLs in European older adults vary with sex, education and self-perceived health over time while simultaneously considering death in the models to eliminate the survival bias. Additionally, it addresses whether there is heterogeneity in sex differences in these trajectories across European countries and contextualises these results according to their cultural and institutional characteristics.

### Previous literature on disability and care dependence of older adults in Europe

In addition to the work of Scheel-Hincke et al. (2020), there is a wide literature focusing on the disability of older adults in Europe. For instance, a previous cross-sectional study estimated the prevalence of disability, defined by the difficulties in performing BADLs, using data from SHARE in 2015 (Jerez-Roig et al. 2018). The authors reported regional differences across European regions (Northern, Central, Eastern and Southern), particularly they reported that older adults with disabilities from East Europe presented the most disadvantaged health profile, followed by the Southern region, and older adults living in the Northern region showed the most advantaged characteristics. Another study reported a considerable prevalence of disability that has been slowly decreasing in the period 2009 to 2017 (Carmona-Torres et al. 2019). Using cross-sectional data from Spain, they also reported that disability was associated with female sex, advanced age, and lower educational attainment, among other risk factors.

In a cross-sectional study, it was reported that older women have worse functioning and higher levels of disability in performing IADLs than men, but disability in performing BADLs was not different between men and women in the USA and in European countries (Crimmins et al. 2011). Moreover, a previous work using cross-sectional data from India showed sex differences in self-reported disability (Bora and Saikia 2015). Besides, older women have a higher number of activity limitations and there was heterogeneity across European countries (Lima et al. 2018).

Sex patterns of difficulties in performing BADLs and/or IADLs may be affected by the different roles of men and women in household activities, and a comparison across different cultures and institutional contexts could provide new insights into this heterogeneity (Millán-Calenti et al. 2010; Sheehan and Tucker-Drob 2019). Hence, differences between men and women may be relevant to inform actions that ensure the quality of life in older adults despite their difficulties and care needs (Beach et al. 2018).

Thorslund et al. (2013) reported that the female advantage in Life Expectancy found worldwide has been narrowing. However, the differences in disability-free life expectancies could still be relevant to understand for how long older adults live with difficulties in BADLs and/or IADLs that may require help (Moreno et al. 2020; Solé-Auró et al. 2015).

Many previous studies addressed differences in disability between older women and men using cross-sectional data. However, there is relatively less previous work using a longitudinal perspective which considers death simultaneously. Nevertheless, some studies have implemented a longitudinal perspective to analyse the health trajectories of older adults. For instance, Amengual et al. (2021) developed a methodology to classify individuals into groups, exploiting health information from panel data they estimated transitions across latent categories (health groups) and death, conditioning on socio-demographic characteristics and current health status. In turn, Arrighi et al. 2017) analysed the socio-economic determinants of transitions across disability and frailty states in European countries and reported an overrepresentation of socio-economically disadvantaged groups in the current cohorts of dependent older adults.

On the other hand, some studies aimed to understand the mix of care used by dependent older adults. For instance, Geerts and Van den Bosch (2012) analysed the transitions between formal and informal care utilisation across nine European countries between the first two waves of SHARE. Their results suggested that, while rates of formal care utilisation continue to differ considerably across European countries, formal care allocation practices are not very different across Northern and Continental European welfare states. In addition, they explored how macro-contextual factors affect transitions in formal and informal care utilisation by older Europeans considering two dimensions in which Long-Term Care (LTC) systems could vary: the degree of familial obligations (cultural dimension), and the existence of universal entitlements to public support, in-kind and/or by means of cash benefits (institutional dimension). Briefly, the authors reported that, in Spain and Italy, there is a high degree of family obligations in the cultural dimension and in the institutional dimension a universal needs-based system is absent or partial. In contrast, in the Netherlands, Sweden and Denmark, widely accessible public services coexist with a cultural dimension characterised by limited family obligations. In Austria and Germany, the needs-based entitlement system coexists with a culture of high levels of family obligations. Finally, the authors identify a medium level of family dependency obligations in France and Belgium, coexisting with a universal needs-based entitlement system in France, while in Belgium this system is either non-existent or partial (Geerts and Van den Bosch 2012). The different welfare states in European countries may facilitate (or not) female autonomy and economic independence from the family (Bambra 2004, 2007) and also it has been reported that less developed social policies and more pronounced socio-economic inequalities are related to higher levels of disability (Wahrendorf et al. 2013).

### Aim and contributions

Although the literature on disability in older adults is extensive, previous research has overlooked transitions to disability and death simultaneously and focused on between-person rather than within-person transitions. A better understanding of trajectories to disability of older adults and how sex, education, and self-perceived health shape these trajectories is crucial to inform care policies designed to improve their quality of life and that of their families. However, our current understanding of disability trajectories in older adults is still limited and notably, most of the previous studies used cross-sectional design and ignored death. The proposed analytical strategy allows to consider the competing risks between transitions to disability and death to draw unbiased inferences and evaluate how sex, education, and self-perceived health change the risks of transitions. Moreover, assessing disability trajectories of older adults is particularly relevant in European countries where population ageing is putting pressure on the sustainability of social and health care systems. Finally, it is important to compare the results across countries, as this can provide new insights into disability trajectories and the role of sex in different institutional and cultural contexts (Geerts and Van den Bosch 2012). As a result, this study aims to analyse the role of socio-demographic characteristics (age, sex, and education) and health condition (assessed by the self-perceived health) on transitions towards states with and without difficulties in ADLs considering simultaneously the transition to death. This analysis considers ten European countries that participated in the SHARE study during the period 2004–2013. Additionally, it aims to address the heterogeneity across these European countries, particularly regarding sex differences in the patterns of transition towards disability and death, relating these results to the institutional and cultural contexts. Furthermore, to assess the multidimensionality of disability, the construct is approached by considering difficulties in different types of ADLs, namely BADLs and IADLs.

This analysis improves knowledge of ageing trajectories to inform long-term care policies, applying a longitudinal perspective which considers death, sex differences and the multidimensionality of disability, and contextualising the results regarding differences in cultural and institutional characteristics of the European countries. In particular, this study aims to investigate whether sex differences in the transition to disability can be explained by survival bias. Thus, the contribution is to better understand disability trajectories during the decade 2004–2013 considering death to avoid survival bias, and focusing on sex and cross-country differences. The analytical approach ensures comparability across European countries in order to contextualise the results in the different cultural and institutional contexts. Finally, this work contributes to analysing the heterogeneity in the role of the associated factors on the difficulties to perform different types of ADLs.

## Methods

### Data

The data come from the SHARE study, a multidisciplinary and cross-national panel database where information on health, socio-economic status and social and family networks of individuals aged 50 or older in Europe has been collected since 2004 across European countries (Bergmann et al. 2019; Börsch-Supan et al. 2013). For each participating country, a separate ethical approval was obtained by the respective ethics committees whenever it was required (for more details on the ethical approvals see: http://www.share-project.org/fileadmin/pdf_documentation/SHARE_ethics_approvals.pdf).

This current study includes data of individuals aged 65 years or older at baseline, from 10 European countries (Austria, Belgium, Denmark, France, Germany, Italy, Netherlands, Spain, Sweden and Switzerland). The information was collected over 4 occasions (waves 1, 2, 4 and 5) covering the period from 2004 to 2013. Waves 3, 6, 7 and 8 were excluded from the current analysis to harmonise longitudinal data across countries: a special questionnaire was used in waves 3 and 7, the Netherlands did not participate in wave 6, and wave 8 was different due to COVID-19 and was 6 years apart from the last wave considered hampering the transition analysis. The total analytical sample for the 10 countries combined is 19,691 individuals. Table 1 presents descriptive characteristics of the study entry of the sample in each country. Only respondents with valid data on the difficulty in performing ADLs (> 99%), education (> 97%) and self-perceived health (SPH) (> 99%) and also with two known states during the period 2004–2013 (> 78%) were included in the analyses. Pairwise deletion was used to handle missing data and no weighting procedures were applied to address loss of follow-up (< 32%). Death status and age at death were retrieved from SHARE data up to wave 7 (2017).

**Table 1:** Descriptive characteristics at study entry (*N* = 19,691)

Country	*N*	Age	Female	High education	Better health	ND B&IADLs	D B&IADLs	ND BADLs	D BADLs	Deaths
Austria	2453	73.26 (6.49)	1407 (57.36)	559 (22.79)	664 (27.07)	1861 (75.87)	592 (24.13)	2123 (86.55)	330 (13.45)	245
Belgium	2493	73.45 (6.87)	1374 (55.11)	592 (23.75)	585 (23.47)	1791 (71.84)	702 (28.16)	2021 (81.07)	472 (18.93)	312
Denmark	1345	72.83 (6.84)	734 (54.57)	387 (28.77)	597 (44.39)	1059 (78.74)	286 (21.26)	1181 (87.81)	164 (12.19)	312
France	2647	74.21 (7.04)	1531 (57.84)	365 (13.79)	381 (14.39)	1975 (74.61)	672 (25.39)	2210 (83.49)	437 (16.51)	328
Germany	1282	71.43 (6.00)	643 (50.16)	332 (25.90)	182 (14.20)	1020 (79.56)	262 (20.44)	1113 (86.82)	169 (13.18)	159
Italy	2054	71.77 (6.08)	1051 (51.17)	88 (4.28)	316 (15.38)	1655 (80.57)	399 (19.43)	1763 (85.83)	291 (14.17)	317
The Netherlands	1573	71.94 (6.35)	816 (51.88)	281 (17.86)	407 (25.87)	1320 (83.92)	253 (16.08)	1432 (91.04)	141 (8.96)	214
Spain	2380	74.24 (7.10)	1294 (54.37)	115 (4.83)	288 (12.10)	1709 (71.81)	671 (28.19)	1906 (80.08)	474 (19.92)	552
Sweden	1873	73.04 (7.22)	969 (51.74)	343 (18.31)	678 (36.20)	1481 (79.07)	392 (20.93)	1640 (87.56)	233 (12.44)	455
Switzerland	1591	73.11 (6.63)	826 (51.92)	175 (11.00)	589 (37.02)	1404 (88.25)	187 (11.75)	1474 (92.65)	117 (7.35)	117
Total	19,691	73.09 (6.77)	10,645 (54.06)	3237 (16.44)	4687 (23.80)	15,275 (77.57)	4416 (22.43)	16,863 (85.64)	2828 (14.36)	3011

Values are sample size and %, or mean and standard deviation for Age. “High education” indicates that the highest educational attainment is the first or second stage of tertiary education according to the International Standard Classification of Education 1997, ISCED-97, coding. “Better health” represents that the person reported an “Excellent” or “Very good” self-perceived health, as opposed to reporting “Good”, “Poor” or “Fair” self-perceived health.

ND, no disability; D, disability; B&IADLs, basic and/or instrumental activities of daily living; BADLs, basic activities of daily living

### Variables and measures

For the purposes of the analysis, a series of dummy variables were derived to account for differences in education (1 = the highest educational attainment is first or second stage of tertiary education according to the International Standard Classification of Education 1997, ISCED-97, coding, 0 = otherwise), sex (1 = Female, 0 = Male) and self-perceived health (1 = declared “Excellent” or “Very good” self-perceived health, 0 = declared “Good”, “Poor” or “Fair” self-perceived health). Education was measured at baseline and self-perceived health at each wave. Age and age at death were measured in years.

*Activities of daily living and the disability state.* Difficulties in performing BADLs and/or IADLs were considered to determine states. Six BADLs were considered: dressing, including putting on shoes and socks; walking across a room; bathing or showering; eating, such as cutting up your food; getting in or out of bed; and using the toilet, including getting up or down, while six IADLs were preparing a hot meal, shopping for groceries, making telephone calls, taking medications, doing work around the house or garden, and managing money, such as paying bills and keeping track of expenses.

At study entry, individuals were classified into two possible states: one represents a state without difficulties in performing ADLs (ND) and the other a state with difficulties in performing any ADLs (D). As information about death becomes available, the individuals were classified in the death state (Death).

Two operationalisations of the D state were assessed. First, the D state was determined when the individual had difficulties in performing at least one BADL and/or IADL, corresponding to a comprehensive disability state which considers different types of ADLs (Millán-Calenti et al. 2010). I refer to this operationalization of D state as “disability state”. Secondly, an individual was classified in the D state when declared to have difficulties in performing at least one BADL, without considering the difficulties in performing IADLs. This second operationalization could be considered a “proxy” of the care dependency state. Like in previous studies, having one or more difficulties in BADLs would probably be associated with the need for help to perform daily activities (Rodríguez-Sampayo et al. 2011) and considers also the hierarchy in the loss of functionality with increasing age (Dunlop et al. 1997; Edjolo et al. 2016). Although it is a “proxy” of care dependence, I refer to this operationalization of the D state as a “dependency state”.

### Statistical approach

**Fig. 1:**
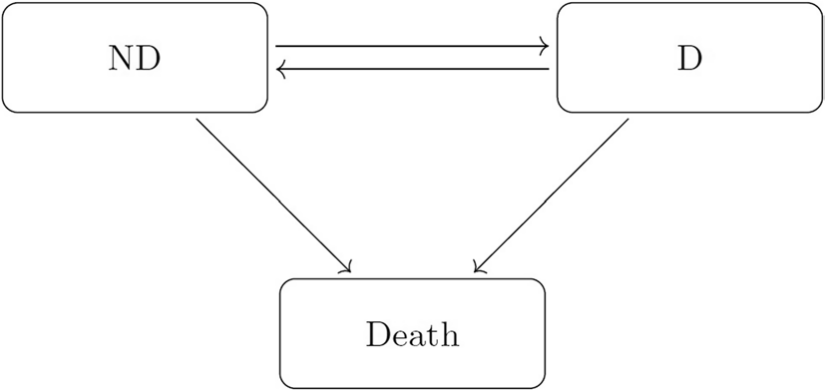
Three-state model for states with and without difficulties in performing ADLs and death. *Notes*: D state was operationalised in two ways: 1-at least one difficulty in performing Basic Activities of Daily Living and/or Instrumental Activities of Daily Living, 2-at least one difficulty in performing Basic Activities of Daily Living. The arrow illustrates transitions modelled over time. ND, no disability; D, disability

Multi-state models (MSM) were used to analyse the transitions across states with and without difficulties in performing ADLs and death (van den Hout 2016b). The MSM model can be defined as an overall fixed-effects model for the process of interest as well as for survival, and when it includes a death state is called a multi-state survival model (van den Hout 2016b). In this model, the probability of transition to disability and the probability of death are estimated simultaneously in such a way that survival bias is considered in the inferences. This parametric approach allows to model transitions among the three states: ND, D and death, and examines the role of risk factors on all transitions simultaneously. As previously mentioned, the D state corresponds to a disability state, with difficulties in performing BADLs and/or IADLs, or a dependency state, with difficulties in performing BADLs, depending on the operationalisation used. See Fig. 1 for a pictorial representation of the three-state model.

Two empirical strategies were implemented. The first strategy assessed simultaneously the role of age, sex, education and self-perceived health on transitions among the three states, with pooled data of individuals from ten European countries. In this strategy, age was included as a covariate on all transitions modelled. Sex, education, self-perceived health and country-specific fixed effects were included in all transitions except for the backward transition from D to ND state. These effects were excluded from the backward transition because it is challenging to assess the recovery from disability with the low number of individuals transitioning from D to ND and the 2-year time windows between observations (Hardy and Gill 2004).

The second strategy focuses on assessing the heterogeneity among these ten European countries with respect to the role of sex in transitions between disability states. In this case, the MSM were estimated for each country allowing the cross-country comparison of the role of sex on the transitions. Hence, age and sex were included as covariates on all transitions, with the exception of the transition from D to ND, due to the same reason as before.

In both strategies, age at death was used to identify death status. In cases where the individual was still alive at the last wave considered (wave 5 in 2013) but the age at death was known from a later wave (wave 6 in 2015 or wave 7 in 2017), it was incorporated in the analysis. Interval censoring was used for transitions between living states because the transition times are not observed in the data (van den Hout 2016b). For instance, the time of onset of disability is known to lie in the time interval defined by two successive observations.

The *msm* package for R (Jackson 2011) was used to estimate the MSM. As a result, the hazard ratios of the covariates over the transitions and their confidence intervals are estimated. The hazard ratios represent the instantaneous transition rate and if they are greater than one indicates a higher instantaneous transition risk conditional on the covariates. In addition, the transition probability across states and its change with age are estimated. At each time point, the probability of transition to disability and to death is estimated as competing event probabilities rather than estimating the transition probability to disability conditional on being alive.

After estimating the MSM, the R package *elect* estimate total and marginal Life Expectancy (LE) (van den Hout 2016a). Total LE is the expected number of years of life remaining at a given age. Marginal LE is obtained for states in which individuals are alive and for a specific state represents the expected number of years spent in that state regardless of the initial state at a given age. The *elect* package fits a multinomial regression model to estimate total and marginal LEs. In this work, the marginal LE obtained for the ND state represents the disability-free LE. Total and disability-free life expectancies were estimated at 65 years for men and women to evaluate the role of sex.

## Results

### Role of age, sex, education and self-perceived health on disability transitions

**Fig. 2:**
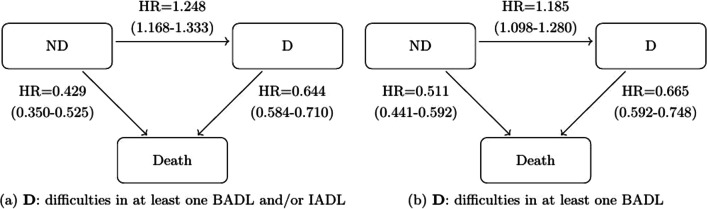
Three-state model with the effect of sex on transitions with pooled data. *Notes*: The figures include the HRs (95% confidence intervals) with pooled data from ten European countries for both operationalisations of D. ND, no disability; D, disability; HR, hazard ratio; BADLs, basic activities of daily living; IADLs, instrumental activities of daily living

**Fig. 3:**
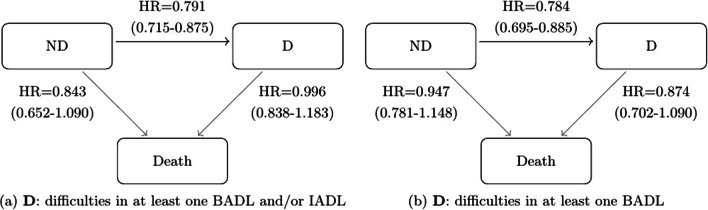
Three-state model with the effect of high education on transitions with pooled data. *Notes*: The figures include the HRs (95% confidence intervals) with pooled data from ten European countries for both operationalisations of D. ND, no disability; D, disability; *HR* hazard ratio; BADLs, basic activities of daily living; IADLs, instrumental activities of daily living

Table 2 shows the effects of covariates on transitions across disability, dependency and death states with pooled data from ten European countries. Figures 2 and 3 depict the hazard ratios for the effects of sex (1 = Female vs. 0 = Male) and education on transitions.

**Table 2:** Hazard ratio and 95% confidence intervals for the effect of covariates on transitions

	Difficulties in BADLs and/or IADLs				Difficulties in BADLs			
	Age	Female	High education	Better health	Age	Female	High education	Better health
ND-D	1.088* (1.081–1.094)	1.248* (1.168–1.333)	0.791* (0.715–0.875)	0.434* (0.395–0.478)	1.085* (1.078–1.092)	1.185* (1.098–1.280)	0.784* (0.695–0.885)	0.436* (0.387–0.492)*
ND-Death	1.085* (1.069–1.101)	0.429* (0.350–0.525)	0.843 (0.652–1.090)	0.530* (0.416–0.677)	1.096* (1.085–1.108)	0.511* (0.441–0.592)	0.947 (0.781–1.148)	0.476* (0.388–0.583)
D-ND	0.949* (0.941–0.957)	–	–	–	0.957* (0.949–0.965)	–	–	–
D-Death	1.079* (1.072–1.086)	0.644* (0.584–0.710)	0.996 (0.838–1.183)	0.629* (0.491–0.804)	1.078* (1.069–1.086)	0.665* (0.592–0.748)	0.874 (0.702–1.090)	0.626* (0.448–0.876)

The table shows the effects of covariates on the risk of transitioning with pooled data from ten European countries. “High education” indicates that the highest educational attainment is first or second stage of tertiary education according to the International Standard Classification of Education 1997, ISCED-97, coding. “Better health” represents that the person reported an “Excellent” or “Very good” self-perceived health, as opposed to reporting “Good”, “Poor” or “Fair” self-perceived health

ND, no disability; D, disability; BADLs, basic activities of daily living; IADLs, instrumental activities of daily living

*5% significant ratio

#### Age

Increasing age was associated with a higher risk of transition to death, either from ND or D states. This result holds for both operationalisations of the D states, specifically when considering difficulties in performing BADLs and/or IADLs (HR = 1.088 CI = [1.081, 1.094]) as well as when considering only difficulties in BADLs (HR = 1.085 CI = [1.078, 1.092]). With regards to the reverse transition, the results suggested that increasing age was associated with a decreasing risk of transitioning from D to ND, also in both operationalisations of D state (see Table 2).

#### Sex

Being female was associated with a lower risk of transitioning from ND or from D to death and a higher risk of transitioning from ND to D for both operationalisations: considering BADLs and/or IADLs (HR = 1.248 CI = [1.168, 1.333]) or only BADLs (HR = 1.185 CI = [1.098, 1.280]) (see Fig. 2).

#### Education

Having a high educational attainment was associated with a lower risk of transitioning from ND to D, when D is a disability or a dependency state. However, a high level of education was not significantly associated with the risk of transitioning to death (see Fig. 3).

#### Self-perceived health

Having reported a better self-perceived health was associated with a lower risk of transitioning from ND to D for both operationalisations and transitioning to death irrespective of the initial state (see Table 2).

### Cross-country comparison of sex differences

#### Sex differences on disability transitions considering death

The results showed great heterogeneity among the European countries regarding the effect of sex on transitions to disability or dependency and death (see Table 3). Also, there were inconsistencies within countries when different operationalisations of the D state were used. When difficulties in performing BADLs and/or IADLs are considered, and D represents a disability state, Spain, Italy, the Netherlands, Austria and Belgium showed a higher risk of transition from ND to D for women than men. However, when considering only difficulties in performing BADLs and the D state represents a dependency state, sex differences are statistically significant only in Spain, Italy and Belgium.

**Table 3:** Hazard ratios and 95% confidence intervals for the effect of sex on transitions by country

Country	Transition ND-D		Transition ND-Death		Transition D-Death	
	Diff. B&IADL	Diff. BADL	Diff. B&IADL	Diff. BADL	Diff. B&IADL	Diff. BADL
Austria	1.365* (1.096–1.700)	1.190 (0.926–1.530)	0.477* (0.258–0.880)	0.609* (0.384–0.968)	0.729 (0.503–1.056)	0.771 (0.496–1.198)
Germany	1.204 (0.936–1.549)	1.057 (0.795–1.405)	0.616 (0.301–1.260)	0.619 (0.340,1.128)	0.692 (0.430–1.114)	0.818 (0.474–1.411)
Sweden	1.086 (0.887–1.331)	0.970 (0.760–1.238)	0.463* (0.271–0.793)	0.565* (0.382–0.834)	0.842 (0.653–1.086)	0.903 (0.660–1.234)
The Netherlands	1.469* (1.155–1.869)	1.201 (0.875–1.648)	0.190* (0.069–0.527)	0.348* (0.205–0.592)	0.637* (0.437–0.929)	0.697 (0.430–1.132)
Spain	1.646* (1.388–1.952)	1.609* (1.323–1.957)	0.362* (0.211–0.620)	0.494* (0.332–0.735)	0.627* (0.503–0.782)	0.592* (0.464–0.755)
Italy	1.562* (1.284–1.901)	1.490* (1.198–1.852)	0.258* (0.114–0.586)	0.364* (0.210–0.630)	0.631* (0.476–0.836)	0.684* (0.494–0.948)
France	1.172 (0.988–1.391)	1.135 (0.932–1.382)	0.292* (0.106–0.801)	0.522* (0.297–0.917)	0.702* (0.537–0.916)	0.720* (0.522–0.994)
Denmark	1.077 (0.839–1.382)	1.045 (0.772–1.415)	0.725 (0.435–1.210)	0.724 (0.500–1.047)	0.653* (0.480–0.889)	0.710 (0.470–1.071)
Switzerland	0.968 (0.730–1.282)	1.101 (0.777–1.560)	0.376* (0.175–0.811)	0.338* (0.193–0.590)	0.482* (0.266–0.874)	0.688 (0.308–1.535)
Belgium	1.242* (1.052–1.466)	1.225* (1.013–1.482)	0.613 (0.315–1.194)	0.590* (0.358–0.974)	0.472* (0.358–0.623)	0.461* (0.331–0.642)

Disability state was determined when the individual had difficulties in at least one BADL and/or IADL. Dependency state was determined as a proxy, when the individual had difficulties in at least one BADL

ND, no disability; D, disability; Diff. B&IADL, difficulties in BADLs and/or IADLs; Diff. BADL, difficulties in BADLs; HR, hazard ratio; BADL, basic activities of daily living; IADL, instrumental activities of daily living

*5% significant ratio

Regarding the transitions to death, most of the countries showed a lower risk of transition for women than men, for both operationalisations and irrespective of the initial state. However, there were some hazard ratios not statistically significant (i.e. for Belgium, Germany and Austria when the initial state is ND and D is a disability state).

Fitting multi-state models also gives the probability of transition among states and how this varies with increasing age (see Figure S.1 and Figure S.2 in supplementary material). The results showed that the probability of transitioning to disability and dependency increases until the age of 70, across countries. This result suggests that after that age, when this probability is stable, the transition to death is more likely. Furthermore, the probability of transition to disability over time is higher than the probability of transition to dependency and, in some countries, statistically significant differences between women and men occur (i.e. Spain and Italy). This result is consistent with the hazard ratios (see Figure S.1 and Figure S.2 in supplementary material).

#### Total and disability-free life expectancies

Table 4 shows the total and disability-free life expectancies for males and females in each country at the age of 65 years. Across all countries total LE at this age is higher for women than men. However, the disability-free LE is very similar for women and men in most countries (except Switzerland or Sweden).

**Table 4:** Life expectancies for male and female participants at 65 years old

Country	Life expectancies in years (95% CI)^a^		Disability-free life expectancy in years (95% CI)^b^	
	Female	Male	Female	Male
Austria	20.87 (20.03,21.72)	18.01 (17.05,18.98)	12.70 (11.86,13.55)	12.74 (11.77,13.70)
Germany	20.98 (19.83,22.13)	18.67 (17.56,19.78)	13.18 (12.04,14.33)	13.13 (12.03,14.24)
Sweden	19.43 (18.61,20.24)	17.19 (16.45,17.93)	14.14 (13.33,14.95)	13.16 (12.42,13.89)
The Netherlands	22.66 (21.51,23.81)	18.11 (17.07,19.15)	14.81 (13.66,15.96)	14.16 (13.12,15.20)
Spain	19.11 (18.48,19.73)	15.97 (15.27,16.66)	11.23 (10.60,11.86)	11.76 (11.07,12.46)
Italy	20.64 (19.87,21.41)	17.69 (16.88,18.50)	12.36 (11.59,13.13)	13.07 (12.26,13.88)
France	22.35 (21.66,23.04)	19.42 (18.65,20.19)	13.05 (12.36,13.74)	12.83 (12.06,13.60)
Denmark	18.80 (17.77,19.83)	16.49 (15.45,17.53)	13.01 (11.99,14.04)	12.42 (11.39,13.46)
Switzerland	24.71 (23.45,25.96)	19.77 (18.67,20.87)	18.16 (16.91,19.42)	15.72 (14.62,16.82)
Belgium	22.94 (22.21,23.66)	18.86 (18.11,19.61)	12.55 (11.82,13.27)	12.54 (11.79,13.29)

D state is a disability state, and was determined when the individual had difficulties in at least one BADL and/or IADL

^a^Total LE in years

^b^LE in state no disability irrespective of where you are at a given age

### Sensitivity analyses

Sensitivity analyses were conducted to assess the robustness of the results regarding the role of sex on the transitions across states in the cross-country analysis. First, we added the covariate indicating high educational attainment to all transitions, with the exception of the transition from D to ND. The results regarding the role of sex on the transitions to disability and dependency were essentially the same as the model that does not include the educational attainment (see Table S.1). Secondly, the models were adjusted by self-perceived health, and some differences emerged in Belgium. In the case of dependency, when only BADLs were considered to determine D state, sex differences were statistically significant in Spain and Italy but not in Belgium. However, for disability state (BADLs and/or IADLs) the results were consistent across the different model specifications (see Table S.2).

Additional analyses were performed by changing the dichotomic variables that measure education and self-perceived health. Tables S.3 and S.4 detail the recoding of the dichotomic variables and showed that results did not change substantially.

## Discussion

This study analysed the role of socio-demographic characteristics and health status in the transition to states with and without difficulties in performing ADLs in ten European countries. A longitudinal perspective was used for the decade 2004–2013. The analytical approach allowed to look at the transition to disability, dependency and death states and how different factors affect them simultaneously.

In pooled country analyses, results showed that the risk of transition varied with age, sex, education and self-perceived health. As expected, increasing age was associated with a higher risk of transition to disability and death. This result was consistent between both measures of disability state, that is when considering difficulties in performing BADLs and/or IADLs or difficulties in BADLs only. Moreover, in the cross-country analyses, the effect of age in all transitions is consistent across European countries and both measures of disability.

Furthermore, in the pooled country analysis, female sex emerged as a risk factor for disability and sex effect in the transition to disability (the risk of transition is 24.8% higher for women) is more than double the effect of age (the risk of transition is 8.8% higher for each additional year). Also, the results showed that female sex is a protective factor for mortality in keeping with previous works (Arrighi et al. 2017; Austad 2006; Bora and Saikia 2015; Cameron et al. 2010; Carmona-Torres et al. 2019; Crimmins et al. 2011; Lima et al. 2018; Oksuzyan et al. 2008; Thorslund et al. 2013). Several factors and a range of explanations have been proposed to understand sex differences in health and mortality. The most common are biological risks, risks acquired through social roles, lifestyle and illness behaviours, and differential healthcare access treatment and use (Oksuzyan et al. 2010). Regarding education, current results suggest that higher educational attainment reduces the risk of transition to disability. However, higher educational attainment does not affect the transition to death. Education has been suggested as a stable indicator of people's social position after young adulthood, representing their material and non-material resources (von dem Knesebeck et al. 2006), ultimately leading to a strong predictor of health in old age (Hoogendijk et al. 2014). Finally, in keeping with previous studies better self-perceived health is associated with a lower risk of disability and mortality, which may be due to its ability to reflect the state of the human organism (Carmona-Torres et al. 2019; Jylhä 2009). However, previous studies have reported that increasing age is associated with worse self-perceived health, but reporting biases would not be as relevant when comparing differences by sex or education (Oksuzyan 2019; Spitzer and Weber 2019).

In the cross-country analysis, it was possible to compare the differences in trajectories towards disability and dependency considering death, in the different cultural and institutional contexts of the ten European countries. The results showed that for some countries the effect of sex on the risk of transition to disability remains significant, while for others the corresponding p values suggested no significant differences between men and women. There were sex differences in the transition to a state with difficulties in performing BADLs and/or IADLs in Spain, Italy, the Netherlands, Austria and Belgium. However, sex differences were not statistically significant when considering the transition to a state with difficulties in performing only BADLs, except in Italy and Spain. The higher risk of transition to the dependency of women in Spain and Italy complements existing cross-sectional findings that less developed social policies and more pronounced socio-economic inequalities are associated with higher levels of disability (Wahrendorf et al. 2013). In addition, welfare states in Spain and Italy may increase the risk of disability and dependency for women, where high levels of family obligations coexist with an absent or only partial universal needs-based social care system (Geerts and Van den Bosch 2012). In other countries, such as Denmark or Sweden, the welfare state could provide gender protection. Thus, these results suggest that welfare state policies may be associated with women’s autonomy (Bambra 2004, 2007) and their risk of disability and dependency.

The different results between disability, measured by the difficulties in performing BADLs and/or IADLs, and dependency, measured by the difficulties in performing BADLs are in line with the literature. Previous analyses reported sex differences in difficulties to perform IADLs but not BADLs (Crimmins et al. 2011). As already suggested, the sex division of household tasks could explain the differences between men and women in performing IADLs, with men not performing this type of activities and therefore not reporting difficulties in performing them (Fleishman et al. 2002; Millán-Calenti et al. 2010; Sheehan et al. 2019; Sheehan and Tucker-Drob 2019). Previous work has reported that some IADLs, such as preparing a hot meal or shopping, are mainly performed by women, who may report higher level of difficulty than men (Sheehan and Tucker-Drob 2019).

On the other hand, no statistically significant sex differences are observed in the risk of transition to dependency in most countries (the Netherlands, Austria, Belgium, France, Germany, Sweden, Denmark and Switzerland), suggesting that the “male–female survival-health paradox” do not hold for dependency state. As already argued, difficulties in BADLs can be considered as an indicator of care dependence and thus of a state in which individuals need help from others (Rodríguez-Sampayo et al. 2011). Sex differences in the transition to dependency, as measured by difficulties in performing BADLs, only emerged in Italy and Spain. In Belgium, the results were not robust to different model specifications, so that sex differences in the transition to dependency cannot be ensured in this country. Despite this, the results for Spain and Italy are concerning. In these countries, universal social care systems are absent or partially developed and their cultural norms have a high level of family obligations to care needs, which usually falls in women unpaid work (Geerts and Van den Bosch 2012). Therefore, care policies should take special account of sex differences in order to decrease the burden of care on (informal) family caregivers.

Current results allowed us to understand how the probability of transition to disability changes with increasing age across countries. This probability increases until 70 years old for all countries and both disability measures. Considering that death is not observed for all the individuals participating in the study, the estimated probability of transition to death increases with age, though it is always lower than one. Similarly, the estimated probability of transition to disability after 70 years old flattens rather than decreases to zero with age. Additionally, in most countries the LE suggests that women live with difficulties and may require daily help from others for longer than men.

The current results are not without limitations. Firstly, the approximation of older adults' dependence was analysed through a proxy measure, due to the lack of information about the need for help to perform ADLs in SHARE data. Thus, these results can only contribute to the understanding of dependence that is generated by the person's functional difficulty in performing daily activities. However, with the available data it is not possible to inform about the level of dependence and the need for help from others. For instance, dimensions related to mental health, depressive symptoms, among others may affect the level of dependence state of older individuals beyond their functional disability. It would be imperative that surveys of longitudinal studies of ageing incorporate questions gathering the need for help in ADLs in order to assess the level of dependence in future analyses. Second, the disability measures are based on self-reported and although they are good for approximating this condition, it would be desirable to complement them with other objective measures (Bravell et al. 2011; Duim and Ferrer 2017).

Transitions to disability, dependency and death are affected by age, sex, educational attainment and self-perceived health status. Heterogeneity across European countries in terms of sex differences in transitions to disability and dependency was evident, although not as widespread when we proxy dependence on the basis of difficulties in performing BADLs. However, there were some countries (Italy and Spain) with sex differences in dependency and with their cultural and institutional contexts gender-sensitive care policies are crucial.

## Supplementary Information

**Additional file 1.**
