# Supplementary material for: Transitions across states with and without difficulties in performing activities of daily living and death: a longitudinal comparison of ten European countries
Source: Eur J Ageing. 2023 May 18;20(1):18. doi: 10.1007/s10433-023-00763-0 (PMC10195131; doi:10.1007/s10433-023-00763-0)
Supplement: Supplementary file 1 — Additional file 1. [file 10433_2023_763_MOESM1_ESM.docx]

## Supplementary material

Figure S.1 Probability of transition to disability state with age for women and men by country


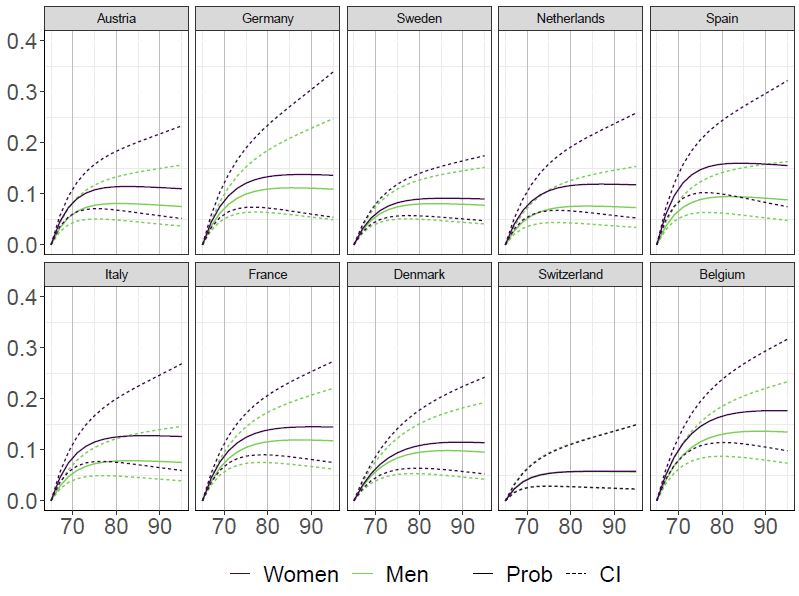


Notes: D state is a disability state, and was determined when the individual had difficulties in at least one BADLs and/or IADLs.

Figure S.2 Probability of transition to dependency state with age for women and men by country


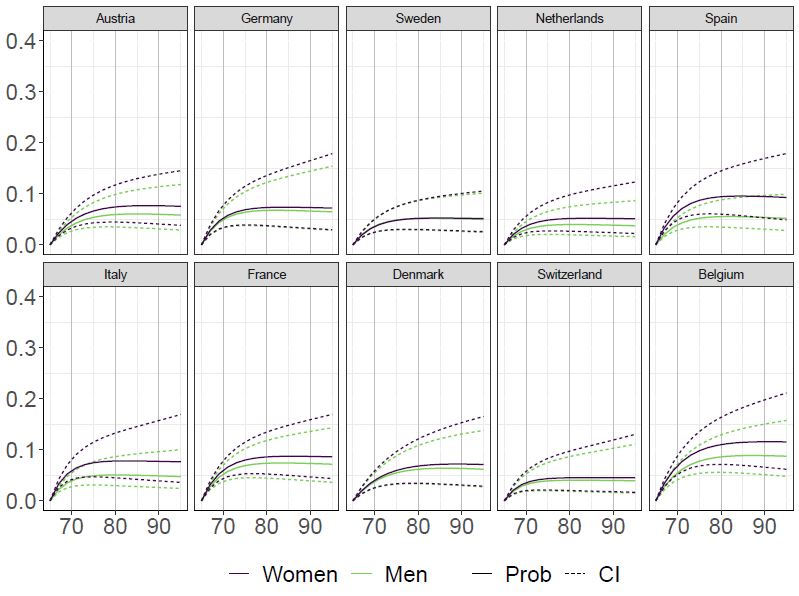


Notes: D state is a dependency state, and was determined when the individual had difficulties in at least one BADLs.

Table S.1 Hazard ratio and 95% confidence intervals for the effect of sex on transitions by country

|  | Transition ND - D | |
| --- | --- | --- |
| Country | Diff. B&IADL | Diff. BADL |
| Austria | 1.342*  (1.073-1.678) | 1.128  (0.873-1.456) |
| Germany | 1.136  (0.876-1.474) | 1.032  (0.768-1.386) |
| Sweden | 1.109  (0.902-1.364) | 0.989  (0.772-1.268) |
| The Netherlands | 1.486*  (1.154-1.913)* | 1.269  (0.898-1.792) |
| Spain | 1.668*  (1.401-1.986) | 1.608*  (1.318-1.963) |
| Italy | 1.558*  (1.279-1.897) | 1.493*  (1.199-1.858) |
| France | 1.119  (0.941-1.331) | 1.075  (0.880-1.312) |
| Denmark | 1.040  (0.809-1.338) | 1.025  (0.756-1.391) |
| Switzerland | 0.928  (0.694-1.240) | 1.097  (0.768-1.566) |
| Belgium | 1.233*  (1.043-1.457) | 1.218*  (1.006,1.475) |
| Notes: The table shows the effect of being female on the risk of transitioning in the model that includes age, sex, and educational attainment. Disability state was determined when the individual had difficulties in at least one BADLs and/or IADLs. Dependency state was determined as a proxy, when the individual had difficulties in at least one BADL. Abbreviations: ND: No Disability; D: Disability; Diff. B&IADL: Difficulties in BADLs and/or IADLs; Diff. BADL: Difficulties in BADLs; HR, hazard ratio; BADL: Basic Activities of Daily Living; IADL: Instrumental Activities of Daily Living. * 5% significant ratio. | | |

Table S.2 Hazard ratio and 95% confidence intervals for the effect of sex on transitions by country

|  | Transition ND - D | |
| --- | --- | --- |
| Country | Diff. B&IADL | Diff. BADL |
| Austria | 1.321*  (1.056-1.654) | 1.095  (0.846-1.418) |
| Germany | 1.154  (0.891-1.495) | 1.036  (0.773-1.390) |
| Sweden | 1.037  (0.841-1.279) | 0.926  (0.719-1.193) |
| The Netherlands | 1.518*  (1.175-1.96) | 1.246  (0.877-1.771) |
| Spain | 1.652*  (1.387-1.969) | 1.588*  (1.301-1.939) |
| Italy | 1.521*  (1.247-1.855) | 1.452*  (1.166-1.808) |
| France | 1.133  (0.952-1.348) | 1.059  (0.867-1.294) |
| Denmark | 1.028  (0.796-1.326) | 1.005  (0.738-1.369) |
| Switzerland | 0.889  (0.666-1.188) | 1.074  (0.753-1.533) |
| Belgium | 1.217*  (1.030-1.439)* | 1.202  (0.993-1.456) |
| Notes: The table shows the effect of being female on the risk of transitioning in the model that includes age, sex, educational attainment, and self-perceived health. Disability state was determined when the individual had difficulties in at least one BADLs and/or IADLs. Dependency state was determined as a proxy, when the individual had difficulties in at least one BADL. Abbreviations: ND: No Disability; D: Disability; Diff. B&IADL: Difficulties in BADLs and/or IADLs; Diff. BADL: Difficulties in BADLs; HR, hazard ratio; BADL: Basic Activities of Daily Living; IADL: Instrumental Activities of Daily Living. * 5% significant ratio. | | |

Table S.3 Sensitivity of “High education” and “Better health” - Hazard ratio and 95% confidence intervals for the effect of covariates on transitions

|  | Difficulties in BADLs and/or IADLs | | | | | | | |
| --- | --- | --- | --- | --- | --- | --- | --- | --- |
|  | Age | Age^+^ | Female | Female^+^ | High education | High education^+^ | Better health | Better health^+^ |
| ND-D | 1.088*  (1.081-1.094) | 1.086* (1.079-1.093) | 1.248*  (1.168-1.333) | 1.226*  (1.147-1.310) | 0.791*  (0.715-0.875) | 0.833*  (0.772-0.899) | 0.434*  (0.395-0.478) | 0.405*  (0.378-0.434) |
| ND-Death | 1.085*  (1.069-1.101) | 1.086*  (1.071-1.102) | 0.429*  (0.350-0.525) | 0.435*  (0.357-0.528) | 0.843  (0.652-1.090) | 0.961  (0.780-1.183) | 0.530*  (0.416-0.677) | 0.492*  (0.406-0.596) |
| D-ND | 0.949*  (0.941-0.957) | 0.952*  (0.944-0.960) | --- | --- | --- | --- | --- | --- |
| D-Death | 1.079*  (1.072-1.086) | 1.082*  (1.074-1.090) | 0.644*  (0.584-0.710) | 0.641*  (0.580-0.709) | 0.996  (0.838-1.183) | 0.920  (0.811-1.044) | 0.629*  (0.491-0.804) | 0.615*  (0.543-0.696) |
| Notes: The table shows the effects of covariates on the risk of transitioning with pooled data from ten European countries. The estimates were obtaining with two different dichotomisations of level of education and Self-perceived health. I present the original estimates and the ones obtained with the different dichotomisation which are indicated with a superscript +. “High education” indicates that the highest educational attainment is first or second stage of tertiary education according to the International Standard Classification of Education 1997, ISCED-97, coding. “High education+” indicates that the highest educational attainment is (Upper) secondary education, Post-secondary non-tertiary education, first or second stage of tertiary education according to the International Standard Classification of Education 1997, ISCED-97, coding. “Better health” represents that the person reported an “Excellent” or “Very good” self-perceived health, as opposed to reporting “Good”, “Poor” or “Fair” self-perceived health. “Better health+” represents that the person reported an “Excellent”, “Very good” or “Good” self-perceived health, as opposed to reporting “Poor” or “Fair” self-perceived health.  Abbreviations: ND: No Disability; D: Disability; BADLs: Basic Activities of Daily Living; IADLs: Instrumental Activities of Daily Living. * 5% significant ratio. | | | | | | | | |

Table S.4 Sensitivity of ‘High education’ and ‘Better health’ - Hazard ratio and 95% confidence intervals for the effect of covariates on transitions

|  | Difficulties in BADLs | | | | | | | |
| --- | --- | --- | --- | --- | --- | --- | --- | --- |
|  | Age | Age^+^ | Female | Female^+^ | High education | High education^+^ | Better health | Better health^+^ |
| ND-D | 1.085*  (1.078-1.092) | 1.082* (1.074-1.089) | 1.185*  (1.098-1.280) | 1.149*  (1.064-1.242) | 0.784*  (0.695-0.885) | 0.829*  (0.758-0.906) | 0.436*  (0.387-0.492)* | 0.410*  (0.379-0.444) |
| ND-Death | 1.096*  (1.085-1.108) | 1.096*  (1.085-1.108) | 0.511*  (0.441-0.592) | 0.507*  (0.439-0.585) | 0.947  (0.781-1.148) | 0.969  (0.827-1.136) | 0.476*  (0.388-0.583) | 0.465*  (0.401-0.538) |
| D-ND | 0.957*  (0.949-0.965) | 0.958*  (0.950-0.967) | --- | --- | --- | --- | --- | --- |
| D-Death | 1.078*  (1.069-1.086) | 1.080*  (1.071-1.089) | 0.665*  (0.592-0.748) | 0.658*  (0.584-0.742) | 0.874  (0.702-1.090) | 0.863  (0.739-1.008) | 0.626*  (0.448-0.876) | 0.618*  (0.527-0.725) |
| Notes: The table shows the effects of covariates on the risk of transitioning with pooled data from ten European countries. The estimates were obtaining with two different dichotomisations of level of education and Self-perceived health. I present the original estimates and the ones obtained with the different dichotomisation which are indicated with a superscript +. “High education” indicates that the highest educational attainment is first or second stage of tertiary education according to the International Standard Classification of Education 1997, ISCED-97, coding. “High education+” indicates that the highest educational attainment is (Upper) secondary education, Post-secondary non-tertiary education, first or second stage of tertiary education according to the International Standard Classification of Education 1997, ISCED-97, coding. “Better health” represents that the person reported an “Excellent” or “Very good” self-perceived health, as opposed to reporting “Good”, “Poor” or “Fair” self-perceived health. “Better health+” represents that the person reported an “Excellent”, “Very good” or “Good” self-perceived health, as opposed to reporting “Poor” or “Fair” self-perceived health.  Abbreviations: ND: No Disability; D: Disability; BADLs: Basic Activities of Daily Living; IADLs: Instrumental Activities of Daily Living. * 5% significant ratio. | | | | | | | | |
